# Novel KPC-2 variants and epidemic ST463 clones underlie ceftazidime/avibactam resistance in carbapenem-resistant *Pseudomonas aeruginosa*

**DOI:** 10.1128/spectrum.02187-25

**Published:** 2025-12-08

**Authors:** Yanyan Hu, Qiaoling Sun, Weiyi Shen, Jingjing Du, Zeng Kaiqi, Rong Zhang

**Affiliations:** 1 Department of Clinical Laboratory, Second Affiliated Hospital of Zhejiang University, School of Medicine, Hangzhou, China; 2 Department of Laboratory Medicine, The First Affiliated Hospital, Zhejiang University School of Medicine, Hangzhou, China; JMI Laboratories, North Liberty, Iowa, USA

**Keywords:** carbapenem-resistant *Pseudomonas aeruginosa*, sequence typing 463, KPC-2 variants, ceftazidime/avibactam

## Abstract

Carbapenem-resistant *Pseudomonas aeruginosa* (CRPA) presents a serious clinical challenge due to limited treatment options. Ceftazidime–avibactam (CZA) is a critical last-resort agent against multidrug-resistant Gram-negative pathogens; however, resistance is emerging, particularly mediated by *bla*_KPC_ variants. In this study, we investigated the prevalence and mechanisms of CZA resistance among CRPA isolates collected from two tertiary hospitals in Hangzhou between 2021 and 2022. A total of 273 non-duplicate CRPA isolates were screened, among which 13.9% (38/273) exhibited CZA resistance (MIC ≥ 16 µg/mL). Whole-genome sequencing revealed that resistant strains were highly resistant to carbapenems, cephalosporins, and aztreonam but retained partial susceptibility to aminoglycosides and polymyxins. ST463 was the predominant sequence type (76.3%), strongly associated with *bla*_KPC-2_ carriage and virulence genes, such as *exoU* and *pldA*. Notably, three *bla*_KPC_ variants—KPC-14, KPC-33, and KPC-86—were identified, all conferring high-level resistance to CZA (MIC > 32 µg/mL). KPC-86 was discovered for the first time in *P. aeruginosa*, embedded in an IS*26*-flanked transposon, suggesting potential for horizontal transfer. Sequential recovery of KPC-86 and KPC-33 from the same patient supports *in vivo* evolution under antibiotic pressure. These findings highlight the evolving threat of CZA resistance in CRPA, driven by *bla*_KPC_ diversification within high-risk clones. Continued genomic surveillance and antimicrobial stewardship are urgently needed to preserve the efficacy of CZA.

**IMPORTANCE:**

Ceftazidime–avibactam (CZA) is one of the few remaining options to treat serious infections caused by drug-resistant *Pseudomonas aeruginosa*. However, its effectiveness is now being threatened by emerging genetic changes that reduce susceptibility. In this study, we investigated CZA-resistant strains and uncovered three variants of the resistance gene *bla*_KPC_, including one—KPC-86—never before seen in *P. aeruginosa*. This variant was located on a mobile genetic element, raising concerns about its potential to spread between bacteria. We also documented the evolution of resistance within a single patient, showing how treatment pressure can drive genetic change. By combining clinical, microbiological, and genomic data, our findings highlight an urgent need for ongoing surveillance and careful antibiotic use to preserve the usefulness of last-resort drugs like CZA. This work informs both clinicians and public health experts on emerging resistance threats.

## INTRODUCTION

*Pseudomonas aeruginosa* is a prominent opportunistic pathogen and a leading cause of nosocomial infections, particularly among immunocompromised patients. These infections, including pneumonia and bloodstream infections, can be severe and are associated with high morbidity and mortality rates, especially when caused by multidrug-resistant strain*s* (1). Treatment of nosocomial infections due to multidrug-resistant *P. aeruginosa* presents significant challenges, primarily due to the limited availability of effective antimicrobial agents (2, 3). Carbapenems have traditionally been considered the cornerstone of therapy for severe infections caused by *P. aeruginosa*; however, the emergence of carbapenem-resistant *P. aeruginosa* (CRPA) has considerably complicated treatment options (4, 5). The sustained high prevalence of CRPA poses a substantial public health threat, further narrowing the already limited range of effective antimicrobial therapies.

Given *P. aeruginosa*’s intrinsic resistance to tigecycline, the remaining therapeutic options for CRPA include ceftazidime–avibactam (CZA), colistin, and amikacin. The 2023 CHINET surveillance report indicated that the resistance rates of *P. aeruginosa* to CZA, colistin, and aminoglycosides were all below 10%. However, both colistin and amikacin are infrequently used as monotherapies due to their significant toxicity profiles. The 2024 Infectious Diseases Society of America guidelines recommend CZA as the first-line treatment for refractory *P. aeruginosa* infections, particularly those caused by CRPA (6).

The global spread of carbapenem-resistant *Klebsiella pneumoniae* (CRKP) harboring the *bla*_KPC_ gene, especially *bla*_KPC-2_, has been well documented (7). The serine active site of KPC is surrounded by four loops: the loop between ⍺3 and ⍺4 helices (Leu102-Ser106), the Ω loop (Arg164-Asp179), the loop between β3 and β4 strands (Cys238-Thr243), and the loop between β5 and ⍺11 helix (Ala267-Ser275). The primary mechanism for CZA resistance in *bla*_KPC_-carrying organisms involves mutations in these loops, which enhance KPC’s affinity for ceftazidime while blocking avibactam binding (8). While CZA has demonstrated clinical efficacy against infections caused by KPC-2-producing CRKP, the increasing use of CZA has led to a rise in CZA-resistant CRKP strains due to *bla*_KPC-2_ mutations. Recent reports have identified similar concerns in CRPA strains (9, 10).

With the widespread emergence of KPC-2-producing CRPA in China (11), it is crucial to evaluate the potential for CZA resistance in CRPA strains when using CZA for treatment. In this study, we investigated the presence of KPC mutants in CZA-resistant CRPA isolates from clinical *P. aeruginosa* samples collected at two large tertiary hospitals in Hangzhou between 2021 and 2022. Additionally, we assessed the risk of CZA resistance in KPC-2-producing CRPA strains under therapeutic pressure, aiming to provide critical insights for cautious clinical management and the rational use of CZA.

## MATERIALS AND METHODS

### Clinical strains

A total of 273 clinical isolates of *P. aeruginosa* were collected from the microbiology laboratories of the First Affiliated Hospital and the Second Affiliated Hospital of Zhejiang University School of Medicine during 2021–2022. Specifically, 202 CRPA isolates were obtained from the First Affiliated Hospital between 2021 and 2022, and 71 CRPA isolates were collected from the Second Affiliated Hospital in 2022. To avoid duplication, repeatedly isolated CZA-resistant strains from the same patient within a 1-month period were excluded. If CZA resistance persisted beyond 1 month, isolates were re-evaluated by susceptibility testing and included as independent samples. Clinical isolates identified as *P. aeruginosa* using matrix-assisted laser desorption/ionization time-of-flight mass spectrometry were collected from the aforementioned hospitals. The antibiotic resistance phenotypes of these isolates were determined using the VITEK 2 Compact automated system with the VITEK 2 AST-N335 card (bioMérieux, France).

### Conjugation assay

Conjugation experiments were performed with three *P. aeruginosa* isolates harboring different KPC variants—#25 (KPC-33), #512 (KPC-14), and #1296 (KPC-86)—using both filter mating and mixed broth methods. Rifampicin-resistant *P. aeruginosa* PAO1 (PAO1^rifR^) and *Escherichia coli* EC600 were used as recipient strains. The detailed experimental method was as described previously (12). For the filter mating method, the mixed culture was incubated on a filter at 35°C overnight. In parallel, the mixed broth method was carried out as previously described (13). Transconjugants were isolated on selective media containing 600 µg/mL rifampicin and 8 µg/mL CZA.

### Antimicrobial susceptibility testing

A total of 273 CRPA isolates were inoculated onto screening plates containing 8 µg/mL of CZA and incubated at 35°C for 18–24 hours. The antimicrobial susceptibility of 38 strains that demonstrated good growth on the CZA screening plates was then tested against 14 commonly used antibiotics using the broth microdilution method, following the manufacturer’s instructions (Zhuhai DL, Shenzhen, China). In brief, we prepared a bacterial suspension equivalent to 0.5 McFarland standard by using sterile cotton swabs to resuspend colonies in sterile normal saline. Subsequently, 50 µL of the suspension was added to Mueller–Hinton (MH) broth and mixed thoroughly. Then, 100 µL of the resulting mixture was transferred into each well of the antimicrobial susceptibility testing panel. The plates were incubated at 37°C for 18–24 hours under aerobic conditions. Results were interpreted according to CLSI guidelines for *P. aeruginosa* (14). Since CLSI does not provide breakpoints for *P. aeruginosa* against cefoperazone/sulbactam, the breakpoints for Enterobacterales were applied. *P. aeruginosa* ATCC 27853 was used as the quality control strain for the susceptibility testing.

### Genome sequencing and genome analysis

For the *P. aeruginosa* strains with a CZA MIC ≥16 µg/mL, genomic DNA was extracted using the HiPure Bacterial DNA Kit, following the manufacturer’s instructions. Whole-genome sequencing was performed on the mgiseq-t7 platform, with paired-end reads of 150 bp. The raw sequencing data were assembled using SPAdes v3.11.1 software (15). The assembled fasta sequences were used to determine the sequence types (STs) of *P. aeruginosa* using the MLST 2.0 server (https://cge.food.dtu.dk/services/MLST/) (16). The PAst 1.0 server was employed to analyze the serotypes of the *P. aeruginosa* strains (https://cge.food.dtu.dk/services/PAst/) (17). The ResFinder 4.1 platform was used to characterize acquired resistance genes in the draft genomes (https://cge.food.dtu.dk/services/ResFinder/) (18). Reference sequences for the virulence genes were sourced from the VFDB (19) databases. The KPC sequences obtained from the sequencing results were compared with those deposited in the GenBank database. Subsequently, for the two *P. aeruginosa* strains carrying KPC-14 and KPC-86, long-read sequencing was performed using the Nanopore MinION platform. As circular assemblies could not be obtained with Unicycler, a hybrid assembly approach using Flye, followed by error correction with Pilon, was employed to generate complete genome and plasmid sequences (20, 21). Finally, we computed single-nucleotide polymorphisms (SNPs) using Harvest Suite version 1.2 (22). Then the results were annotated and visualized by iTOL version 3.0 software (23).

We downloaded the PDC protein sequences of PDC-3, PDC-8, PDC-30, PDC-34, and PDC-60 in the NCBI GenBank database and aligned them with all 38 whole-genome sequenced strains. And ESPript 3.0 was used to visualize the multiple sequence alignment results (24). Furthermore, secondary structural elements in the alignment were annotated using the crystal structure of PDC from PAO1 (PDB ID: 4wz4) as a reference. In addition, we downloaded the reference sequences of *oprD*, *mexR*, *mexA*, *mexB*, *oprM*, *nalC*, and *nalD* from *P. aeruginosa* PAO1 in the NCBI GenBank database and used them to perform comparative analysis against the corresponding genes extracted from all 38 sequenced isolates.

### Stability of the plasmid carrying KPC variants

The stability of plasmids carrying *bla*_KPC_ variants (KPC-33, KPC-14, and KPC-86) was evaluated by determining the frequency of stable plasmids over multiple passages without selective pressure, similar to our previous methodology with slight modifications (12). Due to the failure of conjugation experiments, the original strains were utilized for plasmid stability assessment. Three individual colonies from each original strain were inoculated into 5 mL of LB broth without antibiotics and cultured at 37°C with shaking at 200 rpm. Daily, 5 µL of the overnight culture was transferred into fresh LB broth at a 1:1,000 dilution. The strains were passaged daily for 15 days, with serial dilutions plated on MH agar and MH agar containing 8 µg/mL CZA at 5-day intervals. Colony counts on MH plates were then compared to those on MH plates with 8 µg/mL CZA. To confirm plasmid retention, 10 colonies were randomly selected from each daily plate and subjected to PCR targeting the *bla*_KPC_ gene. The experiment was conducted in triplicate, including both biological and technical replicates.

## RESULTS

### Antimicrobial susceptibility profiles

Following initial screening and MIC confirmation, 38 CRPA isolates (13.92%, 38/273) were confirmed as resistant to ceftazidime–avibactam (CZA, MIC ≥ 16 µg/mL). These CZA-resistant CRPA (CZA-R) strains exhibited high-level resistance to carbapenems, β-lactams, fluoroquinolones, and monobactams. In contrast, they remained largely susceptible to aminoglycosides and colistin (Table 1).

**TABLE 1:** Antimicrobial susceptibility of 38 CZA-R CRPA strains^*a*^

Antibiotics	MIC range (µg/mL)	MIC_50_ (µg/mL)	MIC_90_ (µg/mL)	Susceptibility rate (%)	Intermediate rate (%)	Resistance rate (%)
IPM	4 to >256	>256	>256	0	7.89	92.11
MEM	8 to >256	>256	>256	0	0	100
CAZ	64 to >256	256	>256	0	0	100
FEP	64 and >256	>256	>256	0	0	100
AK	≤0.5 to >64	4	>64	76.32	0	23.68
LEV	≤0.5 to >16	>16	>16	5.26	0	94.74
CIP	0.25 to >8	>8	>8	5.26	5.26	89.47
CO	≤0.25 to 4	1	1	92.11	5.26	2.63
PB	≤0.5 to 2	1	2	86.84	13.16	0
TZP	32 to >256	>256	>256	0	2.63	97.37
SCF	64 to >256	>256	>256	0	0	100
ATM	8 to >128	>128	>128	5.26	5.26	89.47
GM	≤0.5 to >32	32	>32	39.47	2.63	57.89
CZA	16 to >32	>32	>32	0	0	100

^*a*^ IPM, imipenem; MEM, meropenem; CAZ, ceftazidime; FEP, cefepime; AMK, amikacin; LEV, levofloxacin; CIP, ciprofloxacin; CO, colistin; PB, polymyxin B; TZP, piperacillin/tazobactam; SCF, cefoperazone/sulbactam; ATM, aztreonam; GM, gentamicin; CZA, ceftazidime/avibactam.

### Genomic analysis of resistance and virulence genes

Whole-genome analysis of the 38 CZA-R CRPA isolates identified multiple acquired resistance and virulence genes. All strains harbored chromosomally encoded resistance genes, such as *aph(3')-IIb*, *catB7*, *bla*_PDC_, *bla*_OXA-50_ or its variants (*bla*_OXA-486_, *bla*_OXA-488_), and *fosA*. A wide array of acquired resistance genes was detected, including those conferring resistance to aminoglycosides, β-lactams, fluoroquinolones, tetracyclines, sulfonamides, and macrolides. Among these, *crpP* (97.4%), *sul1* (65.8%), and *aac(6')-Ib-cr/Ib3* (44.7%) were the most prevalent. Notably, 94.7% (36/38) of isolates carried carbapenemase genes, with *bla*_KPC-2_ being predominant (80.6%, 29/36), followed by *bla*_IMP-45_ and *bla*_NDM-1_. All *bla*_KPC-2_-positive strains belonged to the ST463 clone.

**Fig 1:**
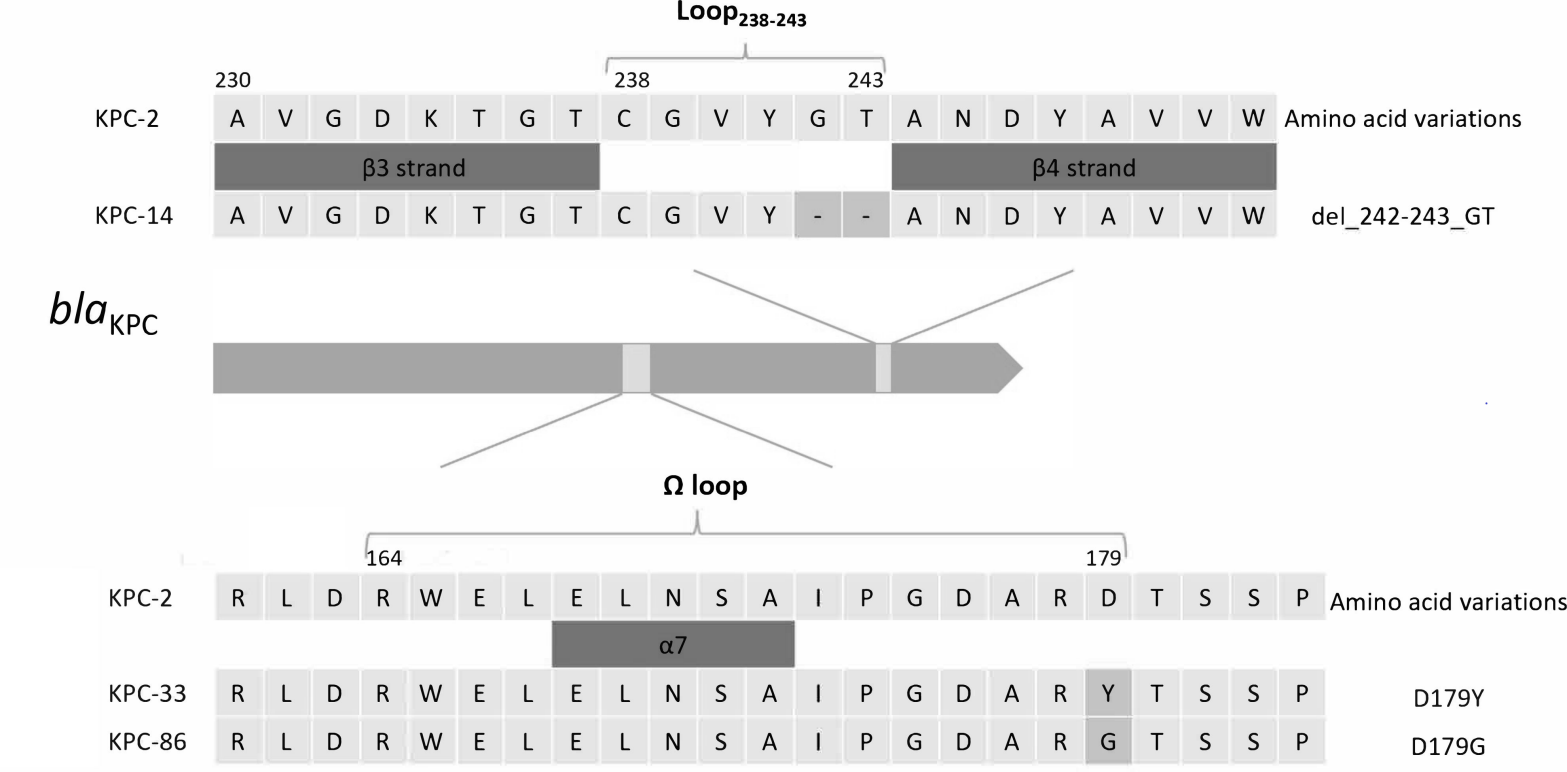
Amino acid sequence alignment of KPC-2 and its variants (KPC-14, KPC-33, KPC-86). Compared to KPC-2, KPC-14 harbors a two-amino-acid deletion at positions Gly242 and Thr243; KPC-33 and KPC-86 exhibit D179Y and D179G substitutions, respectively.

Sequence comparison revealed three KPC variants—*bla*_KPC-14_, *bla*_KPC-33_, and *bla*_KPC-86_—characterized by amino acid substitutions within the Ω-loop or adjacent regions involved in CZA binding. Compared to KPC-2, KPC-14 harbors a deletion of two amino acids at positions 242 and 243 (p.Gly242_Thr243del). KPC-33 and KPC-86 exhibit point mutations D179Y and D179G, respectively (Fig. 1), both of which are located within the conserved Ω-loop region (residues 164–179) and are known to affect substrate binding and inhibitor susceptibility. Strains harboring these variants exhibited high-level resistance to CZA (MIC > 32 µg/mL) and retained resistance to meropenem and imipenem, indicating a shared resistance phenotype (Table 2).

**TABLE 2:** Antibiotic susceptibilities of strains ZY25, ZY512, and ZY1296 (μg/mL)

Isolate	ST	IPM	MEM	CAZ	FEP	AK	LEV	CIP	CO	PB	TZP	SCF	ATM	CZA	GM
ZY1296	463	4	32	>256	>256	>64	>16	>8	1	1	>256	128	64	>32	>32
ZY25	463	4	32	>256	256	>64	>16	>8	1	1	256	128	32	>32	>32
ZY512	463	4	32	>256	>256	2	>16	>8	0.5	1	32	64	>128	>32	1

Virulence gene profiling revealed consistent presence of key determinants, such as *algA*, *exoY*, *fliA*, *lasI*, *rhlA*, *phzM*, and *phzS,* across all isolates. The T3SS effector genes *exoS* and *exoU* were present in 76.3% and 94.7% of strains, respectively, while *pldA* was detected in 94.7%. Importantly, all ST463 isolates co-harbored *exoS*, *exoU*, and *pldA*, indicating a potentially hypervirulent sublineage.

Previous studies have shown that alterations in the Ω-loop structure of the PDC protein may simultaneously affect ceftazidime hydrolysis and avibactam inhibition (25). However, no novel amino acid substitutions, deletions, or insertions were identified within the Ω-loop region or its adjacent contact sites in this study (Fig. S1).

Previous studies have demonstrated that the *oprD* gene plays a crucial role in antibiotic resistance (26). Among the 38 CZA-resistant *P. aeruginosa* isolates analyzed in this study, the most common alteration was a premature stop codon at position 18 (ins42_43TTCC, Ter18), detected in 26 isolates (68.4%). And six isolates (15.8%) had the insertion of the IS*1394* element at nucleotide position 921 (ins921::IS*1394*). Additionally, three isolates exhibited a deletion of nucleotides 697–709, causing a premature stop at codon 234 (Δ697–709 nt, Ter234), and one isolate harbored both a G195A point mutation.

In *P. aeruginosa*, the MexAB-OprM system plays a major role in resistance to carbapenems, and mutations in its repressors, *mexR*, *nalC*, and *nalD*, are also associated with this resistance (27). Compared to the PAO1 reference genome, we observed a V126E mutation in *mexR* (15.8%, 6/38), which has previously been reported not to be associated with increased *mexA* expression (28). In addition, all isolates had G71E and S209R mutations in the *nalC* gene, which were frequently found in the previous research (29). Furthermore, the A37T mutation in *nalD* and the I754V mutation in *mexB* occurred frequently, and no mutations were identified in the *mexA* and *oprM* genes (Table 3).

**TABLE 3:** Genetic variations identified in the *oprD*, *mexR*, *nalC*, *nalD*, *mexA,* and *mexB* genes in *P. aeruginosa* strains

Isolate	*oprD*	*mexR*	*nalC*	*nalD*	*mexA*	*mexB*	*oprM*
ZY957		W	G71E, S209R	W	W	S1041E, V1042A	W
ZY576	ins921::IS1394	V126E	G71E, A145V, S209R	W	W	W	W
ZY410	ins921::IS1394	V126E	G71E, A145V, S209R	W	W	W	W
ZY367	ins921::IS1394	V126E	G71E, A145V, S209R	W	W	W	W
ZY411	ins921::IS1394	V126E	G71E, A145V, S209R	W	W	W	W
ZY277	ins921::IS1394	V126E	G71E, A145V, S209R	W	W	W	W
ZY641	ins921::IS1394	V126E	G71E, A145V, S209R	W	W	W	W
ZR72	G195A, W65Ter	W	G71E, S209R	W	W	W	W
ZY253		W	V40A, G71E, S209R	W	W	W	W
ZY621	Δ697–709 nt, 234Ter	W	G71E, S209R	W	W	I754V	W
ZY732	Δ697–709 nt, 234Ter	W	G71E, S209R	W	W	I754V	W
ZY645	Δ697–709 nt, 234Ter	W	G71E, S209R	W	W	I754V	W
ZR33	ins42_43TTCC, 18Ter	W	G71E, S209R	A37T	W	I754V	W
ZY920	ins42_43TTCC, 18Ter	W	G71E, S209R	A37T	W	I754V	W
ZR36	ins42_43TTCC, 18Ter	W	G71E, S209R	A37T	W	I754V	W
ZY1245	ins42_43TTCC, 18Ter	W	G71E, S209R	A37T	W	I754V	W
ZY512	ins42_43TTCC, 18Ter	W	G71E, S209R	A37T	W	I754V	W
ZY353	ins42_43TTCC, 18Ter	W	G71E, S209R	A37T	W	I754V	W
ZR9	ins42_43TTCC, 18Ter	W	G71E, S209R	A37T	W	I754V	W
ZR7	ins42_43TTCC, 18Ter	W	G71E, S209R	A37T	W	I754V	W
ZY25	ins42_43TTCC, 18Ter	W	G71E, S209R	A37T	W	I754V	W
ZY1296	ins42_43TTCC, 18Ter	W	G71E, S209R	A37T	W	I754V	W
ZY1193	ins42_43TTCC, 18Ter	W	G71E, S209R	A37T	W	I754V	W
ZY1001	ins42_43TTCC, 18Ter	W	G71E, S209R	A37T	W	I754V	W
ZY1083	ins42_43TTCC, 18Ter	W	G71E, S209R	A37T	W	I754V	W
ZY1551	ins42_43TTCC, 18Ter	W	G71E, S209R	A37T	W	I754V	W
ZY1263	ins42_43TTCC, 18Ter	W	G71E, S209R	A37T	W	I754V	W
ZY1264	ins42_43TTCC, 18Ter	W	G71E, S209R	A37T	W	I754V	W
ZY763	ins42_43TTCC, 18Ter	W	G71E, S209R	A37T	W	I754V	W
ZY856	ins42_43TTCC, 18Ter	W	G71E, S209R	A37T	W	I754V	W
ZY759	ins42_43TTCC, 18Ter	W	G71E, S209R	A37T	W	I754V	W
ZY682	ins42_43TTCC, 18Ter	W	G71E, S209R	A37T	W	I754V	W
ZY600	ins42_43TTCC, 18Ter	W	G71E, S209R	A37T	W	I754V	W
ZY793	ins42_43TTCC, 18Ter	W	G71E, S209R	A37T	W	I754V	W
ZY802	ins42_43TTCC, 18Ter	W	G71E, S209R	A37T	W	I754V	W
ZY1564	ins42_43TTCC, 18Ter	W	G71E, S209R	A37T	W	I754V	W
ZY1566	ins42_43TTCC, 18Ter	W	G71E, S209R	A37T	W	I754V	W
ZY1374	ins42_43TTCC, 18Ter	W	G71E, S209R	A37T	W	I754V	W

### Phylogenetic analysis

**Fig 2:**
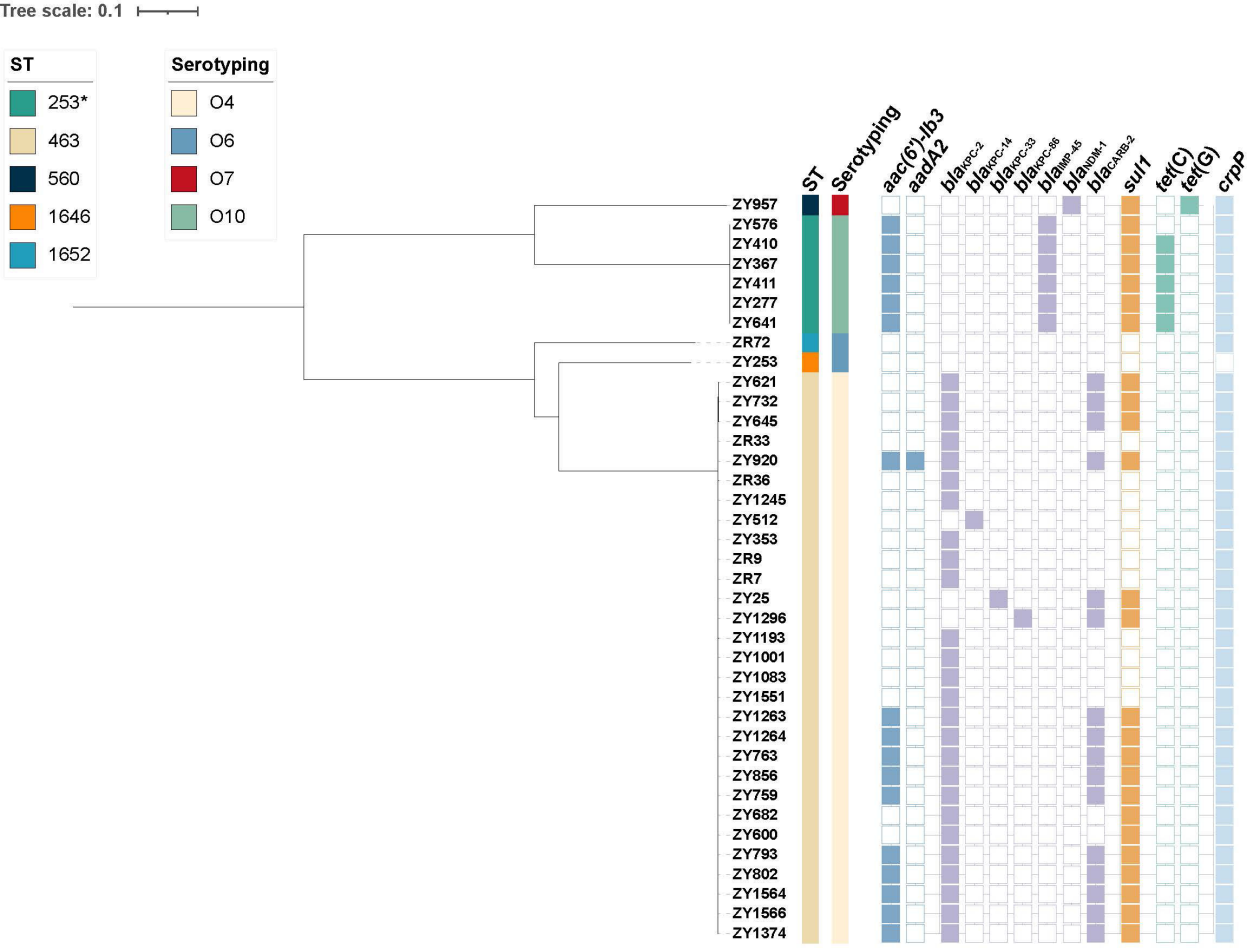
Genomic analysis of 38 CZA-resistant *P. aeruginosa* isolates. A maximum-likelihood phylogenetic tree was constructed using the core genome SNPs. The presence or absence of antibiotic resistance genes is denoted by filled and empty squares, respectively.

We performed core-genome SNP analysis and constructed a phylogenetic tree based on SNP differences (Fig. 2). The results showed that most CZA-resistant isolates clustered within the ST463/O4 clade, indicating the clonal expansion of a high-risk lineage. SNP analysis further revealed that the ST463 isolates were highly genetically related, exhibiting minimal SNP differences. Notably, most ST463 isolates (68.4%, 26/38) possessed the *bla*_KPC-2_ gene, supporting the possibility of clonal transmission events occurring in the clinical setting. Among them, isolates ZY25 and ZY1296, which were collected from the same patient at different time points, differed by only 4 SNPs, suggesting that the CZA-resistant strain likely emerged through *in vivo* evolution under antimicrobial pressure.

### Results of conjugation and plasmid stability

Conjugation experiments were conducted using three *P. aeruginosa* isolates harboring different KPC variants—KPC-33, KPC-14, and KPC-86—against PAO1^rifR^ and EC600 as recipient strains. However, no successful transconjugants were obtained, indicating a failure in the conjugation experiments. Consequently, plasmid stability was assessed using the original *P. aeruginosa* strains. After 15 days of serial passages in the absence of selective pressure, the plasmids carrying *bla*_KPC_ variants (KPC-14 and KPC-86) exhibited stable maintenance within these original strains. No significant differences in colony counts were observed between MH agar plates with or without 8 µg/mL of CZA at each 5-day interval. These findings suggest that the plasmids remained stable and were effectively maintained within the bacterial population, even in the absence of antibiotic pressure. The experiment, conducted with both biological and technical replicates, consistently demonstrated plasmid stability despite the inability to achieve successful conjugation.

### Patient information and clinical analysis of *bla*_KPC-2_ variants

To investigate the relationship between CZA selective pressure and CZA resistance mediated by *bla*_KPC_ variants, we collected and analyzed the clinical information of patients associated with *bla*_KPC_ mutations.

Patient 1: a 17-year-old male with acute lymphoblastic leukemia developed graft-versus-host disease after transplantation and was admitted on 14 September 2021. Due to persistent low-grade fever, he received cefoperazone/sulbactam treatment on 20 September. Following the onset of penile ulcers and a CZA-susceptible CRPA infection on 20 October, treatment was switched to colistin/cefoperazone-sulbactam. On 11 November, fever recurred, and the therapy was changed first to polymyxin B and then to CZA. Despite combination therapy, CZA-resistant CRPA strains (ZY1296 and ZY25) emerged on 29 November 2021 and 4 January 2022, respectively. Due to critical condition and poor prognosis, was discharged on 20 February 2022.

**Fig 3:**
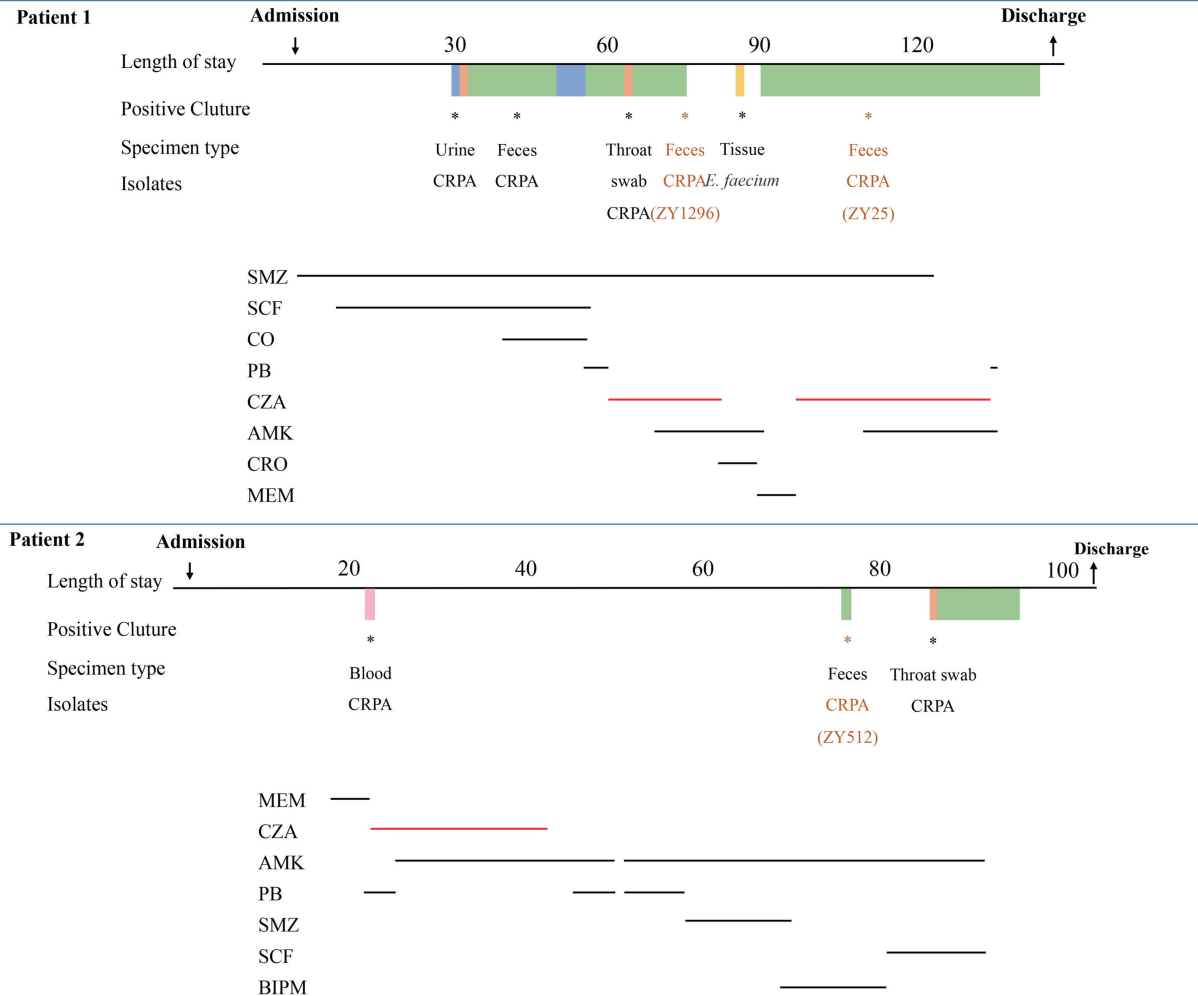
Timelines of the *P. aeruginosa* isolation and the corresponding clinical antimicrobial treatment. SMZ, sulfamethoxazole; CRO, ceftriaxone; BIPM, biapenem; AMK, amikacin; CO, colistin; PB, polymyxin B; CZA, ceftazidime–avibactam; MEM, meropenem; SCF, cefperazone/sulbactam; CRPA, carbapenem-resistant *P. aeruginosa*.

Patient 2: a 55-year-old male diagnosed with acute myeloid leukemia was admitted for chemotherapy on 10 February 2022. Following febrile episodes, CZA-susceptible CRPA was detected in blood and treated with CZA plus amikacin. Following negative blood cultures and normalization of body temperature, the regimen was switched to polymyxin B. Subsequent cultures on 25 April isolated a CZA-resistant CRPA strain (ZY512) from a fecal sample. Ultimately, the patient improved and was discharged on 24 May 2022. The timelines of antibiotic treatment and strain isolation for both patients are shown in Fig. 3.

### Genomic characterization of three KPC-variant *P. aeruginosa*

Among the three identified KPC variants, *bla*_KPC-33_ has been previously reported in *P. aeruginosa*, whereas *bla*_KPC-14_ and *bla*_KPC-86_ are reported here for the first time in this species. To characterize these two variants, we performed third-generation sequencing of the corresponding strains, ZY512 and ZY1296. Both isolates belonged to ST463 and shared the *in silico* serotype O4.

**Fig 4:**
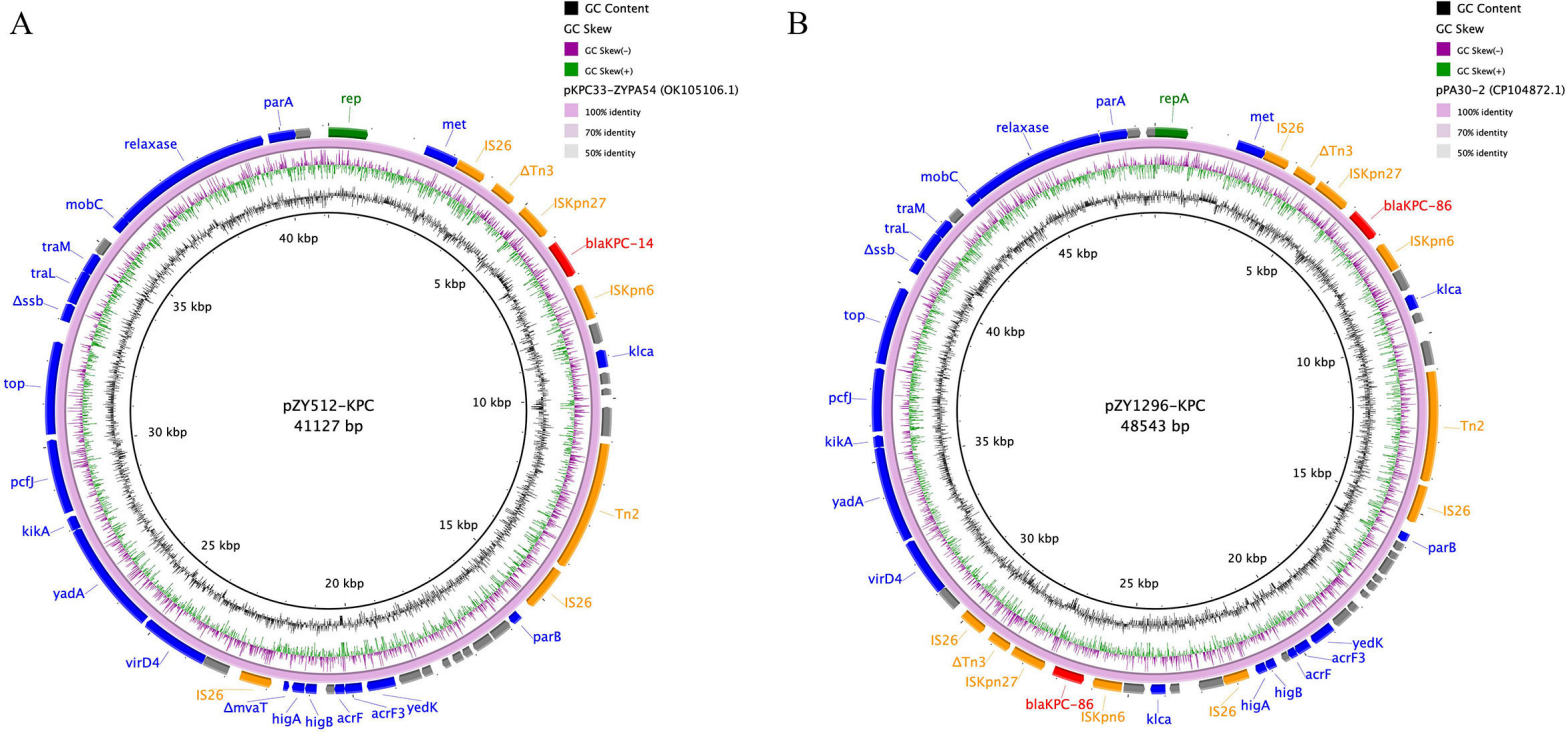
Circular plasmid maps of pZY512-KPC (**A**) and pZY1296-KPC (**B**). Red, orange, green, and blue arrows indicate the antimicrobial resistance genes, mobile elements, repA, and other predicted ORFs, respectively. The names of antimicrobial resistance genes and mobile elements are labeled alongside the corresponding arrows.

The *bla*_KPC-14_-carrying plasmid (pZY512-KPC) and *bla*_KPC-86_-carrying plasmid (pZY1296-KPC) were 41,127 and 48,543 bp in size, respectively (Fig. 4). Comparative plasmid analysis revealed that pZY512-KPC and pZY1296-KPC were highly similar to previously reported plasmids pKPC33-ZYPA54 (GenBank: OK105106.1) and pPA30-2 (GenBank: CP104872.1), showing 100% query coverage and identity.

**Fig 5:**
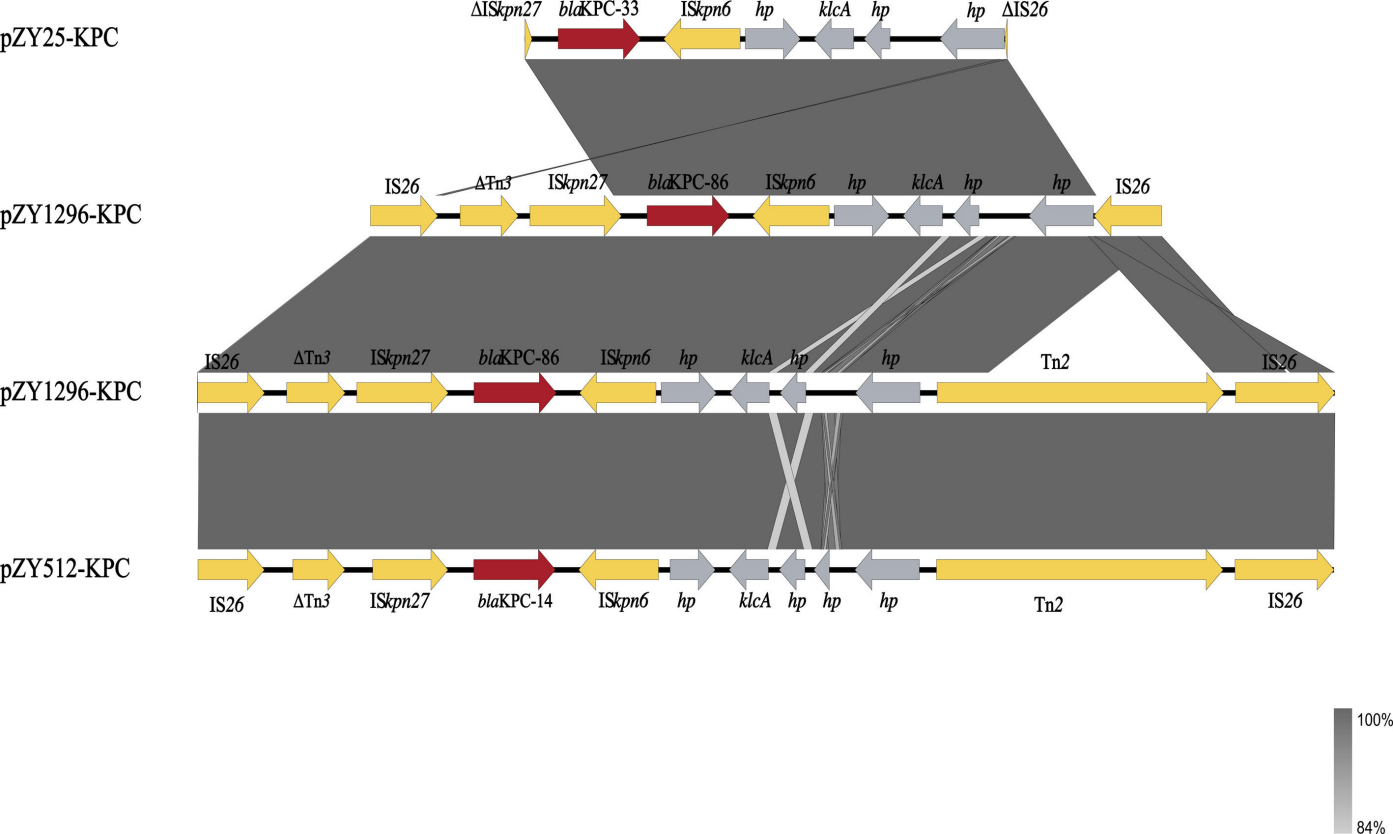
Genetic environment of *bla*_KPC_ genes. Genetic environments of *bla*_KPC-33_, *bla*_KPC-86_, and *bla*_KPC-14_. The arrows indicate the positions and directions of transcription of the different genes. Mobile elements (Tnp and ISs), *bla*_KPC_, and the hypothesis protein are labeled as earthy yellow, wine, and gray, respectively. Δ symbol indicates a truncated gene.

Mobile genetic element analysis indicated conserved genetic environments surrounding the *bla*_KPC_ variants. Both *bla*_KPC-14_ and *bla*_KPC-86_ were flanked by IS*26*-ΔTn*3*-IS*Kpn27-bla*_KPC_-IS*Kpn6*-Tn*2*-IS*26*. An additional copy of *bla*_KPC-86_ in ZY1296 was located within IS*26*-ΔTn*3*-IS*Kpn27-bla*_KPC-86_-IS*Kpn6*-IS*26*, suggesting a potential mobile origin of *bla*_KPC-86_ and may facilitate its dissemination (Fig. 5).

In addition to *bla*_KPC_ variants, ZY512 harbored acquired resistance genes conferring resistance to aminoglycosides, fluoroquinolones, tetracyclines, and chloramphenicol. ZY1296 also co-carried *bla*_CARB-2_. Virulence profiling revealed that both strains encoded key type III secretion system (T3SS) effectors (exoS, exoT, exoU, exoY) and the type VI secretion system (T6SS) effector pldA, as shown in Table 4. These factors have been previously associated with enhanced bacterial fitness and resistance potential (30, 31).

**TABLE 4:** Genetic characterization of KPC-variant harboring *P. aeruginosa* strains

Strains	ST^*a*^	Serotype	KPC type	Resistance genes	Virulence genes
ZY1296	463	O4	KPC-86	*aph(3')-IIb, ant(2'')-Ia, aph(3')-VI, crpP, bla*_OXA-486_*, sul1, bla*_CARB-2_, *bla*_KPC-86_*, bla*_PDC_*, cat*B7*, fos*A	*exoS, exoT, exoU, exoY, pldA*
ZY25	463	O4	KPC-33	*aph(3')-IIb, ant(2'')-Ia, aph(3')-VI, crpP, bla*_OXA-486_*, sul1, bla*_CARB-2_, *bla*_KPC-33_*, bla*_PDC_*, cat*B7*, fos*A	*exoS, exoT, exoU, exoY, pldA*
ZY512	463	O4	KPC-14	*aph(3')-IIb, crpP, bla*_OXA-486_*, bla*_KPC-14_*, bla*_PDC_*, cat*B7*, fos*A	*exoS, exoT, exoU, exoY, pldA*

^*a*^ ST, sequence type.

## DISCUSSION

CZA has emerged as an important therapeutic option against infections caused by carbapenem-resistant Gram-negative bacteria, including *K. pneumoniae* and *P. aeruginosa*. While resistance to CZA has been predominantly reported in KPC-producing *K. pneumoniae*, emerging cases in *P. aeruginosa* are raising increasing concerns regarding its broader efficacy.

In this study, we characterized three *P. aeruginosa* isolates harboring KPC variants associated with CZA resistance, including KPC-14 and KPC-86. While KPC-14 has recently been reported in *P. aeruginosa*, our study provides the first identification of KPC-86 in this species. These findings expand the current understanding of KPC variant distribution and offer new insights into the genetic contexts and potential transmission mechanisms of CZA resistance in *P. aeruginosa*, particularly within the high-risk ST463 clone.

Importantly, all three variants were isolated from fecal samples of hematology patients undergoing chemotherapy or transplantation—individuals characterized by profound immunosuppression and extensive antimicrobial exposure. Moreover, KPC-86 and KPC-33 were sequentially isolated from fecal samples of the same patient, with KPC-86 appearing first and subsequently replaced by KPC-33. This dynamic evolution within the gut suggests a potential within-host selective pressure—likely driven by prolonged CZA exposure—favoring the emergence of distinct KPC mutations. The gastrointestinal tract has been well established as a major reservoir for antimicrobial-resistant organisms, particularly in immunocompromised hosts (32). Moreover, CZA treatment has been implicated in the *in vivo* selection of KPC variants with reduced CZA susceptibility (33), and this finding supports the role of the gut as a hotspot for resistance evolution and potential dissemination.

Whole-genome sequencing and plasmid analysis further revealed that *bla*_KPC-86_ was present in two distinct copies in ZY1296, both embedded within IS*26*-based transposon structures. The duplication of *bla*_KPC-86_, along with the detection of flanking insertion sequences (IS*26*-ΔTn*3*-IS*Kpn27-bla*_KPC-86_-IS*Kpn6*-[±Tn*2*]-IS*26*), suggests that mobile genetic elements, such as IS*26,* play a key role in gene amplification or tandem integration. Such structures have been implicated in the accelerated spread of resistance determinants, potentially enhancing the dissemination capacity of *bla*_KPC_ variants among *P. aeruginosa* (34, 35).

In contrast, *bla*_KPC-14_ was located on a separate plasmid and embedded within a similar IS*26*-flanked transposon structure: IS*26*–ΔTn*3*–IS*Kpn27–bla*_KPC-14_–IS*Kpn6*–Tn*2*–IS*26*. This structure exhibits both similarity and divergence from a recently reported *P. aeruginosa* ST463 strain carrying *bla*_KPC-14_ in dual locations (plasmid and chromosome), where the genetic context was defined as IS*26*–IS*26*–Tn*3*–IS*Kpn27–bla*_KPC-14_–IS*Kpn6*–IS*26* (36). Compared to that, our isolate lacked one upstream IS*26*, carried a truncated Tn*3*, and harbored an additional Tn*2* downstream, indicating a distinct recombination origin. These findings highlight the structural plasticity of IS*26*-mediated transposons and their ongoing role in shaping the evolution of *bla*_KPC_ variants within epidemic *P. aeruginosa* lineages.

Furthermore, the plasmid backbones of pZY1296-KPC (carrying *bla*_KPC-86_) and pZY512-KPC (carrying *bla*_KPC-14_) showed 100% sequence identity to previously reported *P. aeruginosa* plasmids pPA30-2 and pKPC33-ZYPA54, respectively, suggesting structural conservation and possible horizontal transfer events. This implies that despite the genetic diversity observed in the surrounding transposon elements, dissemination of *bla*_KPC_ variants may occur through conserved plasmid vehicles circulating within ST463. These genetic architectures, along with the conservation of plasmid backbones, suggest a shared evolutionary framework for *bla*_KPC_ variants in ST463 strains. Building upon these findings, the detection of *bla*_KPC-14_, *bla*_KPC-86_, and *bla*_KPC-33_ variants in CZA-R *P. aeruginosa* isolates with an identical ST463 background raises concern about the adaptive versatility of this epidemic clone and its capacity to acquire diverse resistance determinants. Although KPC-33 has been previously documented in *P. aeruginosa* (12), our study is the first to report the sequential isolation of KPC-86 and KPC-33 from stool samples of the same patient, suggesting a potential *in vivo* evolution or replacement event. This observation underscores the dynamic nature of resistance development in CRPA under antimicrobial pressure.

In addition, plasmid stability was evaluated in the original host under non-selective conditions. Notably, plasmids harboring *bla*_KPC-33_, *bla*_KPC-14_, and *bla*_KPC-86_ showed high stability over 15-day serial passages without antibiotic pressure. These findings suggest that the observed ceftazidime/avibactam resistance phenotype is unlikely to be attributed to plasmid loss in the absence of selection. The stable maintenance of resistance plasmids may contribute to the persistence and dissemination of CZA-resistant *P. aeruginosa* clones in clinical settings.

Given the co-occurrence of resistance and virulence traits observed in our isolates, particular attention should be paid to epidemic clones with enhanced adaptability, such as ST463. All ST463 isolates in this study, including the three KPC variant strains, harbored *exoU* and *pldA*, two virulence factors previously linked to increased pathogenicity and antimicrobial resistance in *P. aeruginosa* (31, 37). The consistent presence of these genes suggests that ST463 may possess both high-level resistance and virulence, potentially contributing to its persistence and successful spread in clinical settings.

Although KPC variants and metallo-β-lactamases are recognized drivers of CZA resistance, 26 other KPC-2-producing CZA-R-CRPA isolates in our study also exhibited resistance to CZA, without detectable mutations in *bla*_KPC_. We additionally analyzed mutations in the PDC gene, the *oprD* porin gene, and efflux pump-associated genes, such as *nalD*. No notable alterations were detected in PDC, whereas mutations were identified in *oprD* and efflux regulatory genes. However, the specific contribution of these changes to CZA resistance remains unclear, as gene expression levels were not assessed, and detailed mechanistic studies to clarify their roles were not performed, which represents a limitation of our study. However, previous reports have indicated that amplification of the *bla*_KPC_ gene may contribute to reduced susceptibility or resistance to CZA in KPC-producing organisms (38, 39), highlighting the need for close surveillance of CZA-R isolates even when they produce wild-type KPC enzymes.

### Conclusions

In conclusion, our findings highlight the gut as a critical reservoir for resistance evolution in immunocompromised patients and provide the first identification of *bla*_KPC-86_ in *P. aeruginosa*. Although *bla*_KPC-14_ has recently been detected in *P. aeruginosa* from respiratory samples, its identification in our intestinal isolates suggests broader dissemination potential across anatomical sites. Notably, the detection of these KPC variants within the epidemic ST463 clone underscores the genetic plasticity and high-risk nature of this lineage. While ST463 strains harboring *bla*_KPC-2_ remain predominant, the emergence of rare KPC variants with potential resistance implications warrants continuous molecular surveillance to monitor clonal expansion and resistance evolution in CRPA.

## Data Availability

All 38 *P*. *aeruginosa* isolates are deposited at the NCBI website under BioProject ID: PRJNA1263180. The other original contributions presented in the study are included in the article; further inquiries can be directed to the corresponding author.

## SUPPLEMENTAL MATERIAL

Fig. S1 spectrum.02187-25-s0001.pdf Amino acid sequence alignment of the Ω-loop region and adjacent contact sites in PDC.

ASM does not own the copyrights to Supplemental Material that may be linked to, or accessed through, an article. The authors have granted ASM a non-exclusive, world-wide license to publish the Supplemental Material files. Please contact the corresponding author directly for reuse.
