# Supplementary material for: Novel KPC-2 variants and epidemic ST463 clones underlie ceftazidime/avibactam resistance in carbapenem-resistant Pseudomonas aeruginosa
Source: Microbiol Spectr. 2025 Dec 8;14(1):e02187-25. doi: 10.1128/spectrum.02187-25 (PMC12772382; doi:10.1128/spectrum.02187-25)
Supplement: Fig. S1 — Amino acid sequence alignment of the Ω-loop region and adjacent contact sites in PDC. [file spectrum.02187-25-s0001.pdf]

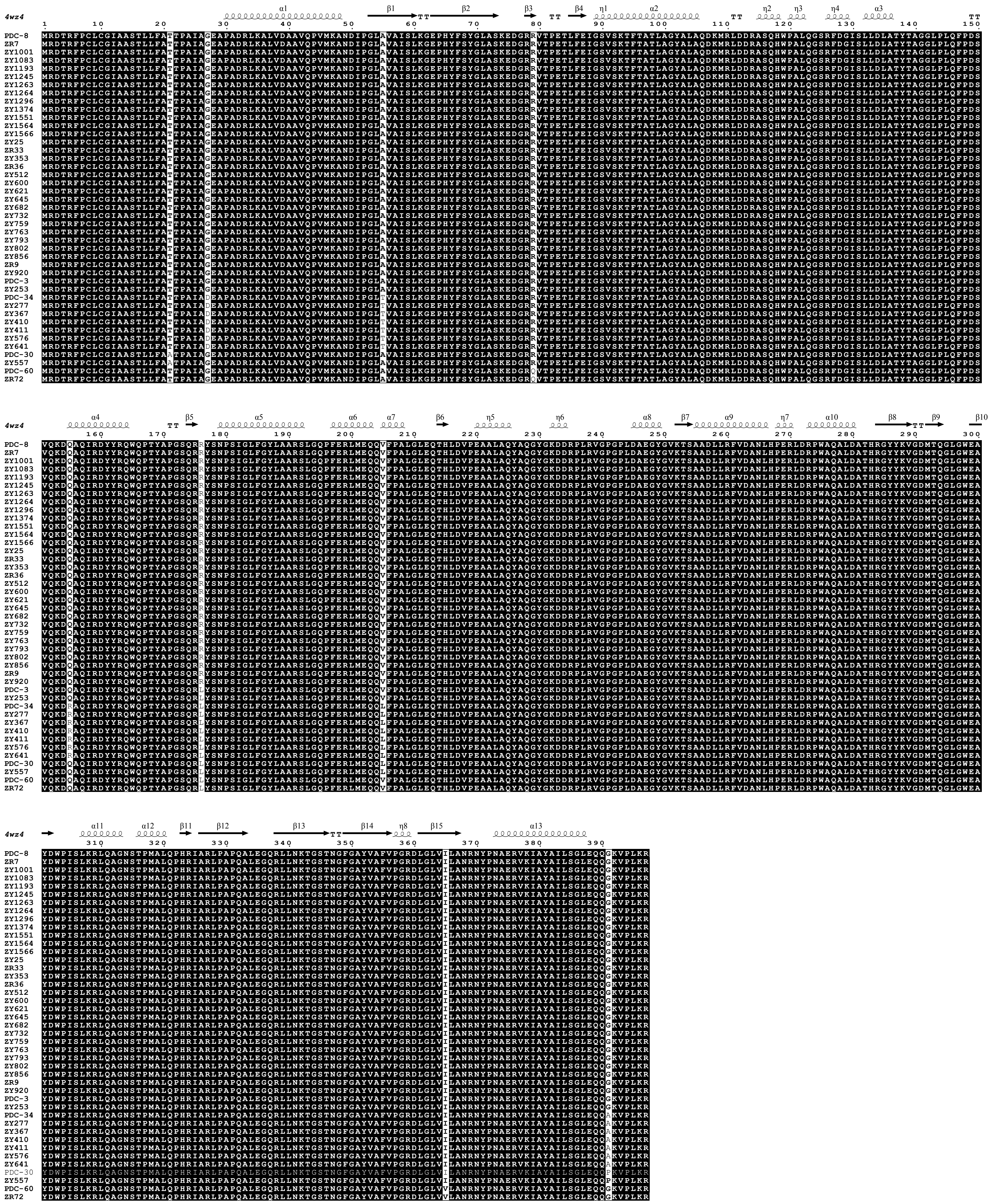

Figure S1. Amino acid sequence alignment of the Ω-loop region and adjacent contact sites in PDC from 38 CZA-resistant *P. aeruginosa* isolates, compared with the reference PDC-3, PDC-8, PDC-30 and PDC-60 sequences.
